# Experimental evidence for anti-metastatic actions of apigenin: a mini review

**DOI:** 10.3389/fonc.2024.1380194

**Published:** 2024-03-07

**Authors:** Hyeon-Muk Oh, Chong-Kwan Cho, Nam-Hun Lee, Chang-Gue Son

**Affiliations:** ^1^ College of Korean Medicine, Daejeon University, Daejeon, Republic of Korea; ^2^ East-West Cancer Center, Daejeon Korean Medicine Hospital of Daejeon University, Daejeon, Republic of Korea; ^3^ East-West Cancer Center, Cheonan Korean Medicine Hospital of Daejeon University, Daejeon, Republic of Korea

**Keywords:** apigenin, cancer, metastasis, anti-metastatic agent, phytochemicals

## Abstract

Cancer metastasis is responsible for the majority of cancer-related deaths. Accordingly, to reduce metastasis remains a vital challenge in clinical practice, and phytochemicals have taken an attention as anti-metastatic agents. Apigenin, a plant flavone, showed anti-cancer effects against in various animal models, moreover its potentials inhibiting tumor metastasis have been reported. Herein, we analyzed the overall features at what apigenin inhibited metastasis and its action modes. We searched for articles in MEDLINE (Pubmed), EMBASE and Cochrane Central Register of Controlled Trials (CENTRAL) through March 2023. Total 6 animal studies presented anti-metastatic effects of apigenin using 5 difference experimental models, while the mechanisms involved modulations of epithelial-mesenchymal transition (EMT), matrix metalloproteinases (MMPs), angiogenesis, and various metastasis-related signaling pathways. This review provides an overall potential of apigenin as a candidate reducing the risk of cancer metastasis.

## Introduction

Cancer metastasis is responsible for the majority of cancer-related morbidity and mortality, accounting for more than 90% of cancer deaths (1). Metastatic cancer is generally more aggressive and difficult to treat, resulting in lower survival rates compared to localized or early-stage cancer (2). Thus, developing effective strategies to target metastasis is crucial for improving survival and overall health of the patient.

On the other hand, many researchers have shown interest in developing anti-cancer agents using natural substances including phytochemicals (3). Epidemiologic studies suggest that a flavones-rich diet can decrease the risk of cancers including lung, breast, and colon cancer (4–6). Among these flavonoids, apigenin has been studied for its anti-tumor properties against various cancer cells, that has been found to 1) inhibit the growth and proliferation of tumor cells along with modulation of signaling pathways including protein kinase B (AKT) and mitogen-activated protein kinase (MAPK) (7–9), 2) induce apoptosis or programmed cell death (10), and 3) inhibit angiogenesis (11, 12), receptively.

Apigenin is a flavone found in a variety of plants such as chamomile, onion, common fruits, and salvia plebeia (13, 14), which partially indicated anti-metastatic effects in preclinical studies (15). In recent years, several studies have systematically reviewed the ‘anti-cancer’ effects of apigenin (16, 17), meanwhile ‘anti-metastatic’ properties of apigenin have not yet been reviewed comprehensively.

This review aims to provide a comprehensive overview of the current knowledge regarding the anti-metastatic properties of apigenin and its potential mechanisms.

## Materials and methods

### Search strategy and selection criteria

Three electronic databases were searched to systematic literature survey including MEDLINE (PubMed), EMBASE and Cochrane Central Register of Controlled Trials (CENTRAL), limited to papers published by March 2023. The search was conducted by combining keywords related to apigenin and metastasis, and its combination.

Because no clinical data exist, the inclusion criteria for the present study were the data evaluating the anti-metastatic effects of apigenin in animal study. We excluded studies that only focused on inhibiting cancer initiation/progression of apigenin or was cell-based data. Articles without full text were excluded.

### Data extraction and analysis

We extracted the following details: name of first author, publication year, country, cancer type (cell line), animal, metastatic model, target organ or site, concentration and duration of apigenin therapy, administration method, primary outcome of the study, and mechanism of actions. Authors have reviewed all included studies carefully, and summarized into table and figure.

## Results

### Characteristics of the included studies

**Figure 1:**
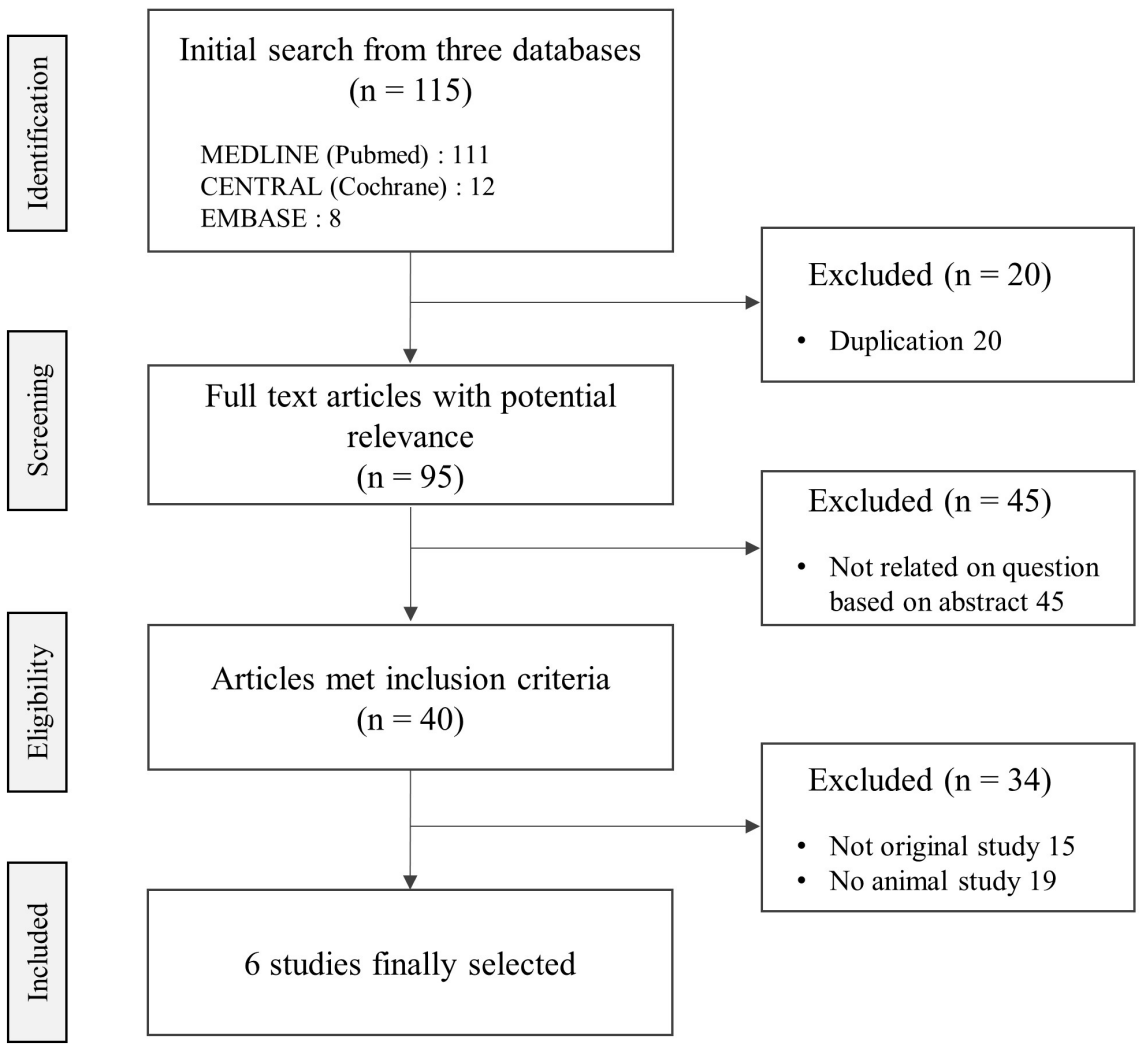
Flow diagram of literature search and selection.

Among the total 40 related articles, 6 studies were finally selected, which presented anti-metastatic effects on 5 mice and 1 rat-based experiments (**Figure 1**). Five different tissue-derived cancer cells (including 1 azoxymethane-injection) were applied for 3 targeted organs/sites (lung, peritoneum, and whole body by bioluminescent imaging), respectively. Five studies were conducted in Asian counties including China (3 studies), Japan (1 study), and Taiwan (1 study), except for 1 study conducted in Italy.

### Anti-metastatic effects of apigenin

All studies confirmed statistically significant anti-metastatic effects by reducing the number of metastatic nodules or colonies at the metastatic sites; lung (2 melanoma and 1 hepatocellular carcinoma), peritoneum (1 ovarian cancer and 1 intestinal adenocarcinoma), and whole body (1 prostate cancer). Apigenin was administered orally at a dose of 150 mg/kg (2 studies), and injected intraperitoneally at a dose of 126.7 ± 123.3 mg/kg (3 studies), subcutaneously at a dose of 1.5 mg/kg (1 study). during 28 ± 14 days on average.

### Anti-metastatic mechanisms of apigenin in tumor tissues

From those results, the main mechanisms explaining anti-metastatic actions of apigenin could be categorized into epithelial-mesenchymal transition (EMT) behavior, matrix metalloproteinases (MMP)-related actions and angiogenesis including several signaling pathways, as summarized in **Table 1**.

**Table 1:** Summary of animal studies on anti-metastatic effects of apigenin.

Author (year)	Cancer type (cell line)	Animal (sex)	Metastatic model	Target organ/site	Drug dose/ Administration/ Duration	Main outcome	Mechanism of actions
Tatsuta et al. (2001) (18)	Intestinal adenocarcinoma	Wistar rat (male)	SC injection of AOM	Peritoneum	1.5 mg/kg, SC 56 days	N. of metastatic colonies^*^	MAPK↓^*^
Piantelli et al. (2006) (19)	Melanoma (B16-BL6)	C57BL/6 (unknown)	Tail vain injection.	Lung	50 mg/kg, IP 14 days	N. of metastatic nodules^*^	VCAM-1↓^*^
He et al. (2012) (20)	Ovarian cancer (OVCAR-3)	Nude mouse (female)	Direct injection to ovaries	Peritoneum	150 mg/kg, PO 28 days	N. of metastatic colonies^*^	AKT signaling pathway↓^*^ MMP-9↓^*^
Qin et al. (2016) (21)	Hepatocellular carcinoma (Bel-7402, PLC)	BALB/c (female)	SC injection to flank	Lung	300 mg/kg, IP 14 days	N. of metastatic nodules^*^	Claudin3↑, E-cadherin↑^*^ Vimentin↓, N-cadherin↓^*^ Snail↓^*^ NF-κB↓^*^
Cao et al. (2016) (22)	Melanoma (B16F10)	C57BL/6 (unknown)	Tail vain injection	Lung	150 mg/kg, PO 24 days	N. of metastatic nodules^*^	MMP2↓, MMP9↓ ^*^ E-cadherin↑, Keratin8↑^*^ Fibronectin↓, N-cadherin↓^*^ Twist1↓^*^ STAT3↓^*^ VEGF↓^*^
Chien et al. (2019) (23)	Prostate cancer	NSG mouse (male)	Intracardiac injection.	Whole-body (bioluminescent imaging)	30 mg/kg, IP 30 days	N. of metastatic colonies^*^	E-cadherin↑^*^ Vimentin↓, N-cadherin↓^*^ Snail↓, Slug↓^*^SPOCK1↓^*^

SC, subcutaneous; AOM, Azoxymethane; MAPK, Mitogen-activated protein kinase; IP, intraperitoneal; VCAM-1, vascular cell adhesion protein 1; PO, per oral; AKT, Protein kinase B; MMP, Matrix metallopeptidase; STAT3, Signal transducer and activator of transcription 3; VEGF, Vascular endothelial growth factor; NSG, NOD scid gamma.

a The concentration indicated the highest concentration used in the experiment.

^*^Statistically significant results are indicated.

The symbol ↓ means upregulate or activate, ↑ means downregulate or suppress.

Apigenin regulated EMT-related molecules such as activations of E-cadherin, claudin3, keratin8, but suppressions of N-cadherin, vimentin, fibronectin, Snail and Slug in 3 different cell lines: liver, melanoma, prostate, respectively. Regarding modulations of MMPs and angiogenesis as the important molecular targets for the anti-metastatic action of apigenin, MMP2 in melanoma cells and MMP9 in melanoma and ovarian cancer cells, and vascular cell adhesion protein 1 (VCAM-1) in B16-BL6 melanoma cell were regulated. In addition, apigenin suppressed other metastasis-favorable molecules including MAPK in intestinal adenocarcinoma cells, AKT in OVCAR-3 ovarian cancer cells, and STAT3 in B16F10 melanoma cells, respectively (**Table 1**).

## Discussion

**Figure 2:**
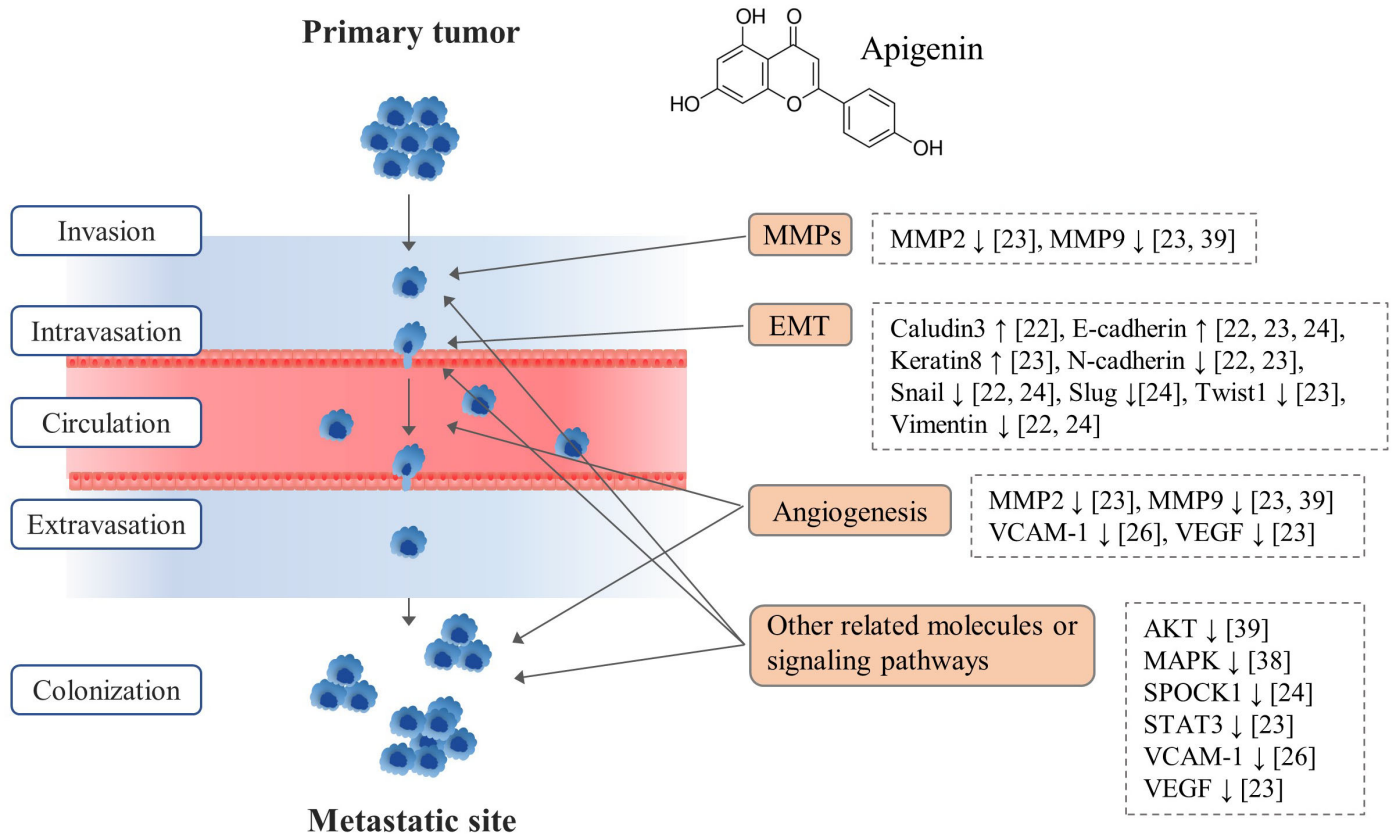
The effect of apigenin in tumor metastatic process.

From 6 apigenin-derived animal studies, apigenin significantly inhibited tumor metastasis by regulating multiple stages of metastatic cascades (**Figure 2**). In fact, tumor metastasis is a complex multistep process, such as firstly primary tumor cell invasion and migration, intravasation, circulation in blood, extravasation, and final colonization in other organs (24).

Due to the heterogeneity and complexity of metastasis in human, there are many obstacles in studying cancer metastasis using animal models. Nevertheless, we aimed to produce a valuable reference for apigenin-derived anti-metastatic drug studies. In our results, five of six studies targeted apigenin-derived anti-metastatic effects against peritoneal-metastasis from ovarian cancer including intestinal tumor and lung-metastasis of melanoma and hepatocellular carcinoma (**Table 1**). This is thought to reflect the pattern of cancer metastasis in humans because ovarian-peritoneum metastasis and the lung metastasis from other sites are the most common in human (25, 26).

From our data, the molecular actions of apigenin’s anti-metastatic effects mainly involved the modulations of EMT, angiogenesis, and related signaling pathways (**Figure 2**). EMT plays an important role in cancer cell metastasis by conferring invasive and migratory capabilities, affected by various molecules such as ZEB family, E-cadherin, N-cadherin (27). As shown in our results, apigenin regulated EMT-related molecules like Claudin3, E-cadherin, Keratin8, N-cadherin, Snail, Slug, Twist1 and Vimentin as reported by 3 animal studies (21–23). In addition, angiogenesis is a critical process not only for primary tumor growth but also for metastasis, enabling colonization of metastatic tumor cells in distant organs (28). Apigenin exhibits anti-angiogenic properties by inhibiting the angiogenic factors such as VEGF, SPOCK 1, VCAM-1 thereby impeding the establishment of secondary tumor sites (19, 22, 23) (**Figure 2**). Moreover, dysregulation of AKT and MAPK signaling pathways is associated with increased metastatic potential (29), which is modulated by apigenin as described in our results.

Although not presented in our study, the immune response and tumor microenvironment are also important for the cancer metastasis. Apigenin has been shown to regulate immune response by modulating programmed cell death 1 (PD1)/programmed cell death ligand 1 (PD-L1) expression in cancer cells (30). Another finding suggested that apigenin increased CD4/CD8 T cells and decreased T regulatory cells in pancreatic cancer cell (31). The synergistic effects of apigenin with standard cancer therapies such as chemotherapy and radiation therapy were also reported (32). Combined use of apigenin can sensitize cancer cells with paclitaxel through suppressing reactive oxygen species (ROS) activity, emphasizing its potential as an adjuvant therapy to enhance treatment efficacy (33). Furthermore, apigenin can alleviated chemotherapy-induced toxicity such as nausea, vomiting, fatigue, and immunosuppression, improving the overall well-being and quality of life in cancer patients (34). The synergistic effect of apigenin with conventional cancer treatment, particularly chemotherapy, offers a promising approach to enhance treatment efficacy while mitigating chemotherapy-induced toxicity.

Apigenin is found in various fruits (orange, grape), vegetables (parsley, lettuce), and herbs (chamomile, peppermint) (35), and then *salvia plebeia* may be of particular interest due to the containing high quantify of apigenin and other medicinal activities (36). S*alvia plebeia* has a long history of traditional medical use in East Asian countries including Korea and China, which has been used to treat various ailments, including respiratory diseases, fever, inflammation, and gastrointestinal disorders (36). Additionally, salvia plebeia contains not only apigenin but also other active compounds such as luteolin, hispidulin, nepetin (14). The multiple bioactive compounds found in salvia plebeia could contribute to its potential medicinal properties and make it an interesting subject of research. From animal study, apigenin was observed to have a long half-life time (91.8 h), suggesting the slow metabolism in the body (37).

Regarding the toxicity of apigenin, it is generally considered as safe and well-tolerated under consuming with a balanced diet (38). Likewise, animal studies have reported no significant adverse effects or mortality even at high doses (39). However, animal studies may not always directly translate to human responses, and further research is needed to establish the safety profile in humans.

The current study has some limitations. First, most studies used different strains and tumor metastatic models, which may vary in their susceptibility to cancer and response to apigenin. Second, while animal studies provide valuable insights into the biological effects of apigenin on metastasis, there are inherent differences between animal models and human biology. In order to apply findings from animal studies to human cancer patients, it is important to consider species-specific differences in metabolism, physiology, and tumor biology.

In conclusion, this review comprehensively supports the potentials of apigenin as a herbal-derived candidate for preventing or inhibiting cancer metastasis, which the underling mechanisms might involve the EMT and angiogenesis.

Considering the encouraging results from our review, future research should aim to bridge the gap between preclinical findings and clinical applications of apigenin in cancer metastasis. Clinical trials that evaluate the efficacy and safety of apigenin in cancer patients, particularly those with metastatic disease, are crucial to validate the translational potential of our findings and pave the way for the development of novel anti-metastatic therapies in the field of oncology.

## Author contributions

H-MO: Writing – review & editing, Writing – original draft, Visualization, Investigation, Formal analysis, Data curation. C-KC: Writing – review & editing, Writing – original draft, Supervision, Resources, Project administration, Methodology, Conceptualization. N-HL: Writing – review & editing, Writing – original draft, Visualization, Supervision, Resources, Methodology, Funding acquisition, Formal analysis. C-GS: Writing – review & editing, Writing – original draft, Visualization, Validation, Supervision, Methodology, Conceptualization.
